# The phosphoric acid extract of fresh biochar and its compound aqueous solutions promoted tobacco plant growth by regulating nutrient-related microorganisms in rhizosphere soil

**DOI:** 10.3389/fmicb.2025.1601567

**Published:** 2025-05-22

**Authors:** Xixi Liu, Lifang Wang, Guowei Li, Chuantao Xu, Yike Li, Yong Peng, Jinping Pu, Yanfen Xie, Yunkun Chen, Zefan Liu, Fuzhao Nian, Di Liu

**Affiliations:** ^1^College of Tobacco Science, Yunnan Agricultural University, Kunming, China; ^2^Luzhou Branch Company, Sichuan Province Tobacco Company, Luzhou, China; ^3^Qujing Branch Company, Yunnan Province Tobacco Company, Qujing, China

**Keywords:** biochar acid extract, compound aqueous solution, tobacco, rhizosphere soil, soil nutrients

## Abstract

**Introduction and methods:**

To further explore the biochar–soil–plant interaction mechanisms, rice husk biochar and root-promoting solutions were used to prepare biochar extracts and compound aqueous solutions, to study the regulation of biochar acid extract and its compound water agent on the growth of tobacco, as well as the effects of the main nutrient content (nitrogen, phosphorus, potassium), microbial abundance, and functional diversity in the rhizosphere soil of tobacco.

**Results:**

The results showed that the application of different concentrations of biochar acid extract and its compound aqueous enhanced tobacco plant dry matter accumulation, improved soil pH, increased available nitrogen and organic matter, and reduced potassium and phosphorus availability. Moreover, the effect of the compound water agent treatment group on soil nutrients was greater than that of the biochar acid extract treatment group. In terms of microorganisms, the application of biochar acid extract and its compound aqueous increased the relative abundance of *Sphingomonas*, *Vicinamibacteraceae* bacterial genus, *Mortierella*, *Penicillium* fungal genus, and functional bacteria such as chemoheterotrophs, aerobic chemoheterotrophs, and saprotrophs, promoting the complexity and interconnectivity of soil microbial networks.

**Discussion:**

In summary, the application of biochar acid extract and its compound aqueous solutions improve soil nutrients by enhancing the structure, diversity, and functional groups of the soil microbial community, thereby promoting tobacco growth. These results not only provide a theoretical basis for the interaction mechanism between biochar, soil, and tobacco, but also provide certain theoretical support for the development of biochar related fertilizers.

## 1 Introduction

Biochar has the characteristics of low density, high specific surface area, and strong stability, and has a wide range of applications in agricultural production (Ping et al., 2021). At the macroscopic level, applying biochar to soil improves soil structure via physical mechanisms, alters key soil element content through chemical processes, and enhances the microbial environment through biological interactions, thereby promoting plant growth. However, at microscopic and molecular levels, the specific biochar components mediating these effects and their underlying mechanisms remain unclear (Yang et al., 2019). Consequently, understanding the organic small molecular compounds present on the surface of biochar has become a critical area of research. Studies indicate that certain volatile organic compounds produced during biochar pyrolysis can act as germination stimulants and biopesticides (Light et al., 2008), and their active ingredients can improve crop germination rates and seedling resilience (Light et al., 2010). Additionally, the washing and cooling processes in biochar production generate substantial amounts of extract, which contain soluble organic substances and can be used as plant nutrients or growth stimulants (Bian et al., 2019). Research confirms that biochar extract enhances seed germination and seedling growth, improves microbial community structure and functional diversity in rhizosphere soil, and regulates soil nutrient content (Lou et al., 2015; Sun et al., 2017).

At present, the preparation of biochar extracts is usually done using water, acid, or alkaline solutions. However, biochar itself is alkaline and mainly composed of insoluble organic small molecule compounds. Preparing extracts with water or alkaline solutions may result in lower concentrations of small molecule compounds dissolved (Zhu et al., 2025). Previous studies have shown that when biochar from the same source is extracted using water, acid, and alkali solutions, the extraction solution prepared using an acidic solution has the highest organic carbon content (Hui et al., 2022). So far, research on the application of biochar extract in tobacco cultivation and production is relatively weak, and its impact mechanism on tobacco growth and soil nutrients is still unclear. Therefore, this study used phosphoric acid to prepare biochar acid extract (BAE) and explored the effects of different concentrations of BAE on tobacco growth, soil nutrients, and microorganisms, providing theoretical support for the use of biochar in tobacco cultivation and production.

In recent years, root-promoting agents have been widely utilized in tobacco cultivation to enhance root vitality, dry matter accumulation, and the quality of flue-cured tobacco leaves (Tianbao et al., 2021; Xia et al., 2023). However, excessive application of these agents can result in adverse effects, including soil compaction and acidification, reduced soil fertility, and disruptions to soil microbial community structures (Xia et al., 2023; Jie et al., 2023). Microorganisms are essential to soil ecosystems, providing critical services such as enhancing soil fertility and plant productivity, while constituting one of the most diverse community structures in ecosystems (Damien et al., 2021). Previous research demonstrated that the combined application of biochar and root-promoting agents significantly improves the root growth of tobacco seedling and enzymatic activity associated with root structure, promoting tobacco growth and quality (Bitao et al., 2020; Jing-Yi et al., 2021; Sales et al., 2020). Furthermore, the use of biochar extract is beneficial to the growth of rice seedlings (Yang et al., 2019), also improve soil organic matter and available nitrogen content, and increase the abundance of dominant bacterial phyla, while boosting microbial *α*-diversity (Li C. et al., 2024). In addition, soil microorganisms can also regulate soil pH, total carbon, and total nitrogen levels, thereby supporting the growth of plants (Yanfen et al., 2023). Despite these advancements, studies on the combined use of BAE and root-promoting agents in tobacco cultivation remains limited, and the mechanisms underlying their effects on tobacco growth and soil nutrients are not well understood. This study aims to develop new compound aqueous solutions based on root-promoting agents and BAE. It will investigate the effects of BAE and these compound solutions on tobacco growth, soil physicochemical properties, microbial communities, and functional diversity within the rhizosphere micro environment. Therefore, the study seeks to clarify the interrelationships among tobacco, soil nutrients, and microorganisms, providing a theoretical foundation for further exploration of root-promoting products and organic small molecular compounds in tobacco cultivation.

## 2 Materials and methods

### 2.1 Experimental field and tobacco variety

The experiment was conducted from April to September, 2023 at the tobacco-planted area in Luzhou City, Sichuan Province (longitude 105.6°E, latitude 28.1°N), situated in a region characterized by a typical Subtropical humid monsoon climate with an average annual temperature of 17.5°C~18.5°C and an average annual rainfall of approximately 1,000~1,200 mm. According to the Chinese classification system for Quaternary Red Clay, the soil is classified as typical yellow soil. The agrochemical properties of the soil were as follows: pH 6.19, organic matter 24.5 g/kg, the alkaline hydrolyzed nitrogen 109.3 mg/kg, available phosphorus 32.5 mg/kg, and available potassium 149.6 mg/kg. The flue-cured tobacco variety was Yunyan 87, supplied by Luzhou Tobacco Company.

### 2.2 Preparation of biochar phosphoric acid extract and its compound aqueous solutions

The acidic extraction of fresh biochar was prepared by mixing fresh rice husk biochar (Pyrolyzed under anaerobic conditions by heating up to 400°C at a rate of 10°C per minute and maintaining it for 2 h, Its physicochemical properties are as follows: pH 9.28, specific surface area 27.17 m^2^/g, pore size 189.53 nm, and pore volume 17.13 cm^3^/mg) with an extracting solution of 0.02 M phosphoric acid at a ratio of 1:25 (1 g biochar to 25 mL phosphoric acid solution). The mixture was agitated at 25 ± 2°C and 100 rpm on a rotary shaker for 24 h (Chai et al., 2024). The resulting supernatant was filtered using a 0.22 μm organic nylon microporous filter membrane with a circulating vacuum pump to obtain the filtrate for further experiments.

To prepare the compound aqueous solutions, the root-promoting agent (2 g naphthylacetic acid, 1.5 g sodium nitrophenolate, 0.2 g indole-3-acetic acid, 0.2 g metalaxyl, 3 g sucrose, 0.2 g amino-ethoxyvinylglycine, and 2.9 g zinc sulfate) (Rohilla et al., 2015) was dissolved in 20 kg of the acidic extraction of biochars. The prepared acidic extraction of biochars and compound aqueous solutions were divided into three aliquots and diluted with deionized water to obtain solutions of 0, 10, and100 times dilution.

### 2.3 Experimental design and implementation

A total of seven treatments were established in the field experiment, each consisting of three replicates with 15 plants per replicate. Each plant was supplied with 50 g of specialized tobacco compound fertilizer (N: P_2_O_5_: K_2_O = 10:15:25). 30 days after the seedlings were transplanted, three concentration gradients of acidic extraction of biochars and compound aqueous solutions were applied to the root systems of the tobacco plants, with each plant receiving 500 mL. The seven experimental treatments were as follows: a control group without the addition of acidic extraction of biochars or compound aqueous solutions (CK); the treatment with acidic extraction of biochars added (S0); acidic extraction of biochars diluted of 10-fold (S10); acidic extraction of biochars diluted of 100-fold (S100); compound aqueous solutions treatment group (W0); compound aqueous solutions diluted of 10-fold (W10); and compound aqueous solutions diluted of 100-fold (W100).

### 2.4 Sample collection

Agronomic traits (number of leaves, plant height, internode length, stem girth、maximum leaf length and maximum blade width) were assessed during the resetting stage (30 days after the tobacco seedlings transplanted), vigorous growing stage (60 days after the tobacco seedlings transplanted), and upper leaves mature stages (120 days after the tobacco seedlings transplanted) of the tobacco plants following the tobacco industry standard (YC/T 142-2010 “Survey Method for Tobacco Agronomic Traits”) (Yang et al., 2024).

Samples were collected at the upper leaves mature stage, after removing the surface soil, the entire root system was extracted, and the rhizosphere soil sample was collected by gently shaking the roots, with five duplicate samples for each treatment. Some samples were quick frozen in liquid nitrogen for 30 min, then stored at −80°C for microbial diversity analysis. The remaining samples were naturally dried and sieved through a 2 mm sieve to remove impurities.

Before the lower leaves of tobacco plants were harvested, the whole tobacco plant was collected, root system was rinsed with running water. The whole plant samples were divided into root, stem, and leaf sections, and these parts were briefly heated at 105°C for15 min, then dried to a constant weight at 70°C, furtherly, calculating the root-to-shoot ratio.

### 2.5 Sample analysis

Air-dried soil samples are sieved through a 1 mm sieve to measure agrochemical properties, soil pH was measured using the water extraction method, organic matter (OM) content was assessed via the potassium dichromate titration method, available nitrogen (AN) content was quantified using the alkaline diffusion method, available phosphorus (AP) content was measured using the molybdenum-antimony colorimetric method, available potassium (AK) content was determined by the ammonium acetate-flame photometric method, and soil bulk density (SBD) was analyzed using the core sampler method (Zhao et al., 2025).

The E.Z.N.A.TM Kit was used to extract soil microbial genomic DNA, which was then analyzed via 0.8% agarose gel electrophoresis and quantified using a NanoDrop 2000. The 16S rRNA gene’s V3-V4 region (Qiao et al., 2022), corresponding to the complete bacterial community, was amplified using 338F/806R primers. Simultaneously, the fungal ITS1 gene was amplified using ITS1a and ITS1b primers (Usyk et al., 2017). After purifying the PCR products (Vazyme Biotech Co., Ltd. Nanjing, China), they were quantified using a BioTek FLx800. Paired-end sequencing was performed using the Illumina NovaSeq platform and NovaSeq 6000 SP Reagent Kit (500 cycles) (Parsortix, Shanghai, China). The QIIME2 2019.4 platform was used for microbiome bioinformatics analysis (Tianjiao et al., 2022), which included primer trimming, quality filtering, denoising, merging, and chimera removal.

### 2.6 Data analysis

Independent sample t-tests were conducted to analyze agronomic traits, biomass, and soil agrochemical properties under different treatments. All data are presented as means ± standard error (SE), and visualizations with Origin 2021 software. The sequence data analysis primarily utilized QIIME2 and R packages (v3.2.0). A network analysis of the top 50 abundant bacterial and fungal communities was conducted using microbial ASVs to examine interspecies relationships. Gephi visualized (v0.10.1) was employed to explore symbiotic patterns in soil microbial communities based on strong (*p* > 0.6) and significant (*p* < 0.01) correlations. Node sizes corresponded to connectivity with other nodes, and coloration reflected genus classification. Distance-based redundancy analysis was to investigate the relationship between soil microbial community structure and agrochemical properties. All statistical analyses were conducted using SPSS 22.0 software.

## 3 Results

### 3.1 Effects of BAE and the compound aqueous solutions on tobacco plant biomass

**Figure 1:**
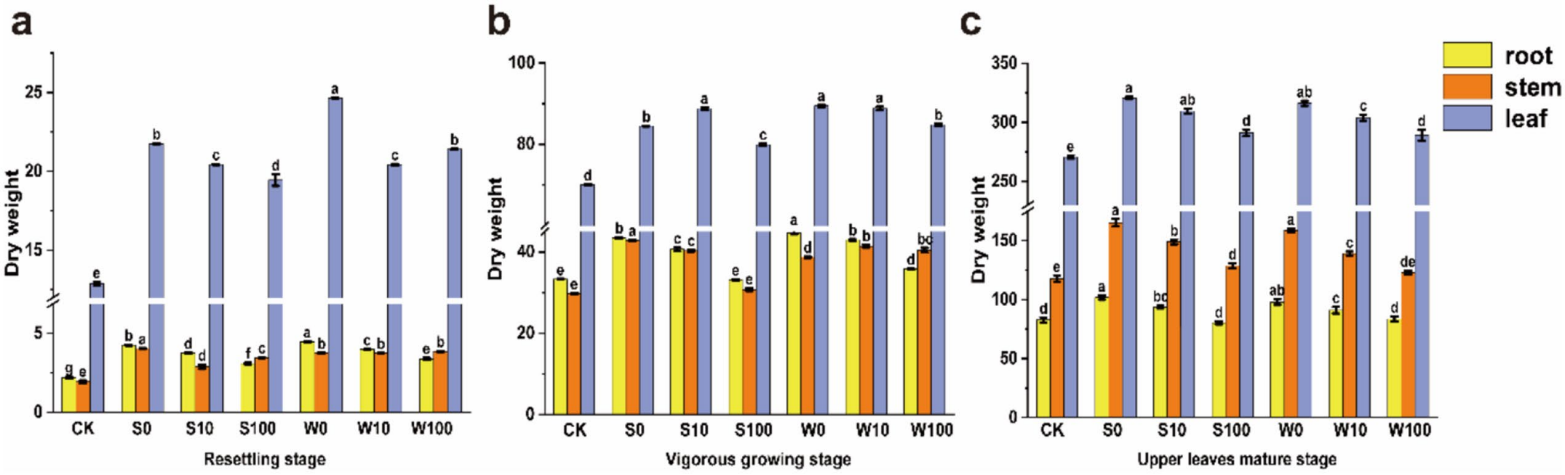
The biomass of flue-cured tobacco plant at resettling stage **(a)** vigorous growing stage **(b)** and upper leaves mature stage **(c)** under different treatments. Using one-way analysis of variance, different lowercase letters indicated significant differences between treatments (*p* < 0.05).

The application of BAE and compound aqueous solutions affected the agronomic traits and biomass of the treated tobacco plants, as shown in Figure 1 and Supplementary Table S1, which enhanced agronomic traits and biomass during the resettling, vigorous growing, and upper leaves mature stages, compared to the control group (CK), and the promoting effects gradually diminished with decreasing concentrations of BAE and compound aqueous solutions. The compound aqueous solution demonstrated a more promoting effect than the BAE, with the most pronounced enhancement observed during the maturity stage. These results indicated that both BAE and its compound aqueous solutions positively promote tobacco biomass and agronomic traits throughout the tobacco plant growth stages, with higher concentrations producing better effect. Furthermore, the compound aqueous solutions consistently outperformed the BAE of the promoting effects.

### 3.2 Effects of BAE and the compound aqueous solutions on the agrochemical properties of the rhizosphere soil

The effects of the treatments on the agrochemical properties of the soil are summarized in Table 1. Compared to the control group (CK), the application of BAE and its compound aqueous solutions significantly increased soil pH and SBD, which were higher with greater dilution of the acid extract and compound solution. The application of undiluted (0-fold dilution) BAE and its compound aqueous solutions significantly increased soil OM content. However, as the concentration decreased, soil OM content gradually declined, reaching levels lower than those in CK at a 100-fold dilution. The compound aqueous solutions also significantly increased soil AN content, with the promoting effect positively correlated with concentration, whereas the opposite was observed for the BAE. Additionally, both BAE and compound aqueous solutions significantly reduced soil AK and AP levels, with reductions becoming more pronounced at lower concentrations. In summary, the application of BAE and compound aqueous solutions notably improved soil pH, OM, AN, and SBD. While the positive effects on soil pH and SBD decreased with dilution, the opposite trend was observed for OM content of the BAE and compound aqueous solutions. All treatments also caused significant reductions in soil AP and AK, with effects more pronounced at higher concentrations.

**Table 1:** Physicochemical properties of rhizosphere soil of flue-cured tobacco under different treatments.

Treatment	pH	OM (g/kg)	AN (mg/kg)	AP (mg/kg)	AK (mg/kg)	SBD (g/cm^3^)
CK	6.34 ± 0.04d	40.86 ± 0.30e	163.74 ± 0.44e	66.15 ± 0.09c	275.48 ± 0.17a	0.88 ± 0.00f
S0	6.90 ± 0.06c	50.36 ± 0.06b	159.89 ± 0.33f	65.97 ± 0.14b	235.59 ± 0.15e	0.92 ± 0.01de
S10	7.06 ± 0.03b	43.85 ± 0.45d	160.70 ± 0.41f	55.24 ± 0.03e	248.48 ± 0.07c	0.93 ± 0.00 cd
S100	7.19 ± 0.10a	38.68 ± 0.69f	195.08 ± 0.39b	51.02 ± 0.11f	216.64 ± 0.41f	0.96 ± 0.00a
W0	7.04 ± 0.09b	57.05 ± 0.10a	206.12 ± 0.41a	68.60 ± 0.28a	254.44 ± 0.14b	0.91 ± 0.01e
W10	7.07 ± 0.01b	46.13 ± 0.35c	182.05 ± 0.43c	56.47 ± 0.28d	237.36 ± 0.07d	0.94 ± 0.00bc
W100	7.15 ± 0.05ab	39.26 ± 0.10f	172.16 ± 0.36d	46.21 ± 0.06 g	207.99 ± 0.18 g	0.95 ± 0.01ab

Using one-way analysis of variance, different lowercase letters indicated significant differences between treatments (*p* < 0.05). OM, organic matter; AN, available nitrogen; AP, available phosphorus; AK, available potassium; SBD, soil bulk density.

### 3.3 Effects of BAE and the compound aqueous solutions on soil microbial community composition

#### 3.3.1 Impact on soil microbial community structure

**Figure 2:**
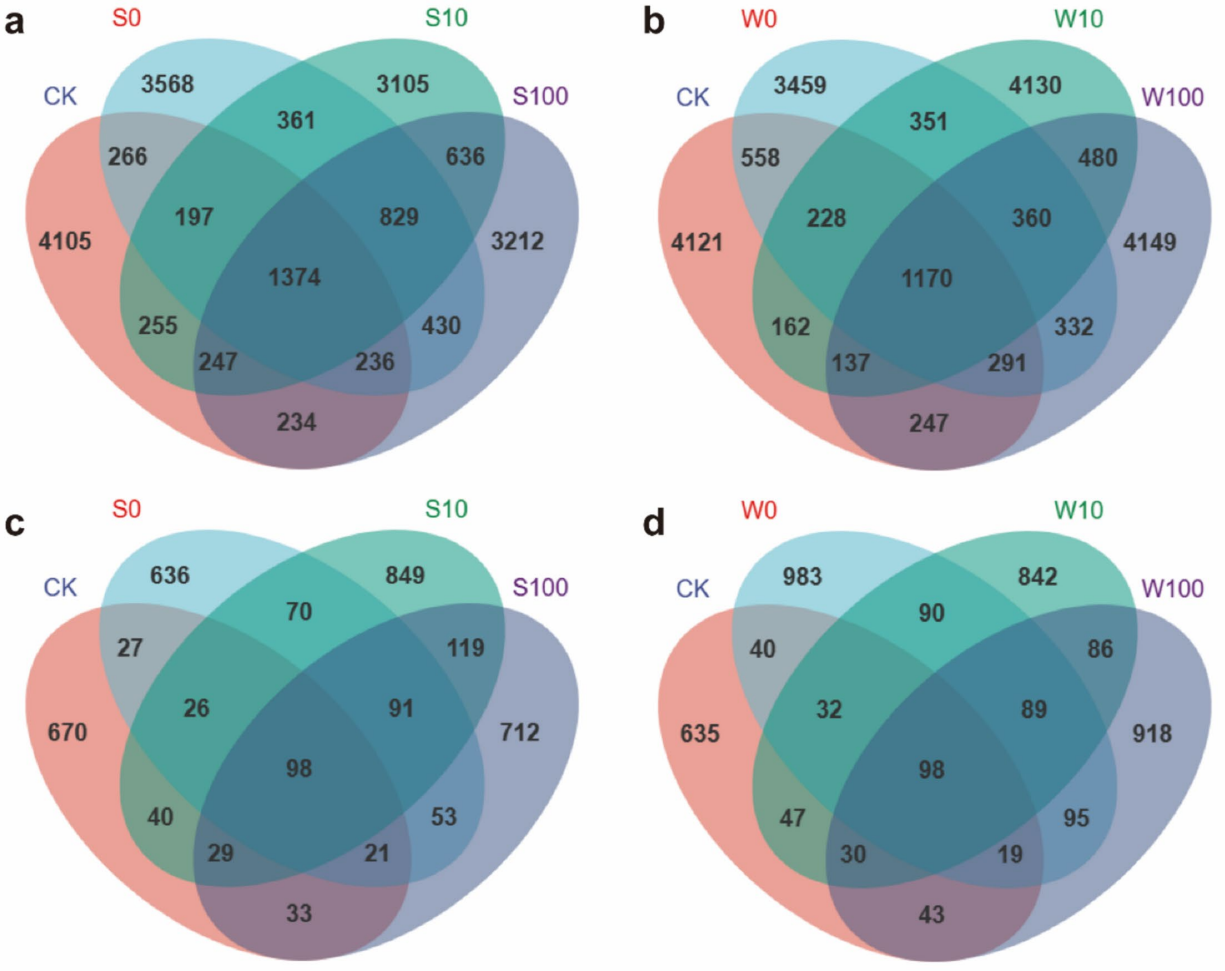
Venn plot of bacteria **(a,b)** and fungal **(c,d)** in rhizosphere soil of flue-cured tobacco plants under different treatments. (ASV level) Each circle represents a sample, the number of circles and the overlapping parts of the circles represent the number of ASVs common to the samples, and the numbers without overlap represent the number of ASVs unique to the samples.

The analysis of bacterial and fungal ASVs (amplicon sequence variants) in the rhizosphere soil of tobacco identified a total of 22,657 bacterial ASVs and 4,851 fungal ASVs. In the control group (CK) and the S0 treatment, the unique bacterial ASVs numbered 4,105 and 3,568, respectively, while the unique fungal ASVs were 670 and 636, respectively. For the S10 and S100 treatments, the unique bacterial ASVs were 3,105 and 3,212, and the unique fungal ASVs were 849 and 712, respectively (Figures 2a,c). Similarly, in the CK and W0 treatments, the unique bacterial ASVs were 4,121 and 3,459, respectively, while the unique fungal ASVs were 635 and 983. For the W10 and W100 treatments, the unique bacterial ASVs were 4,130 and 4,149, while the unique fungal ASVs were 842 and 918, respectively (Figures 2b,d). These results indicate that applying BAE reduced the number of unique bacterial ASVs in the rhizosphere soil of tobacco while increasing the number of unique fungal ASVs, with a more pronounced effect on fungal ASVs.

**Figure 3:**
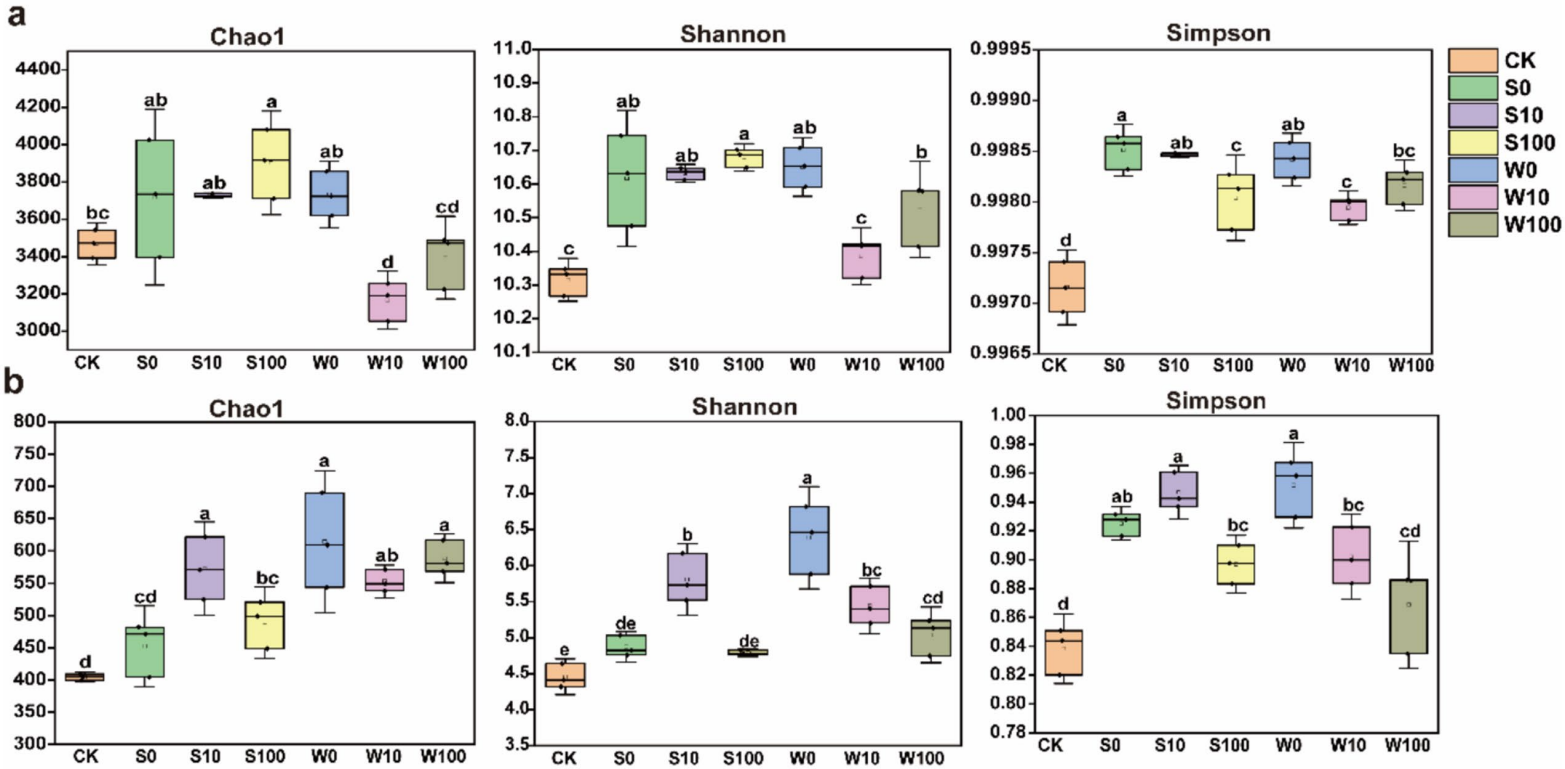
Chao1, Shannon, and Simpson index of bacteria **(a)** and fungal **(b)** in rhizosphere soil of flue-cured tobacco under different treatments. Using one-way analysis of variance, different lowercase letters indicated significant differences between treatments (*P* < 0.05).

The application of BAE and its compound aqueous solutions changed the *α*-diversity of both bacterial and fungal communities (Figure 3; Supplementary Table S2). Specifically, the Chao1, Shannon, and Simpson indices for fungi reached their highest values at a 10-fold dilution. While bacterial α-diversity indices also increased, the changes were not statistically significant. Compared to the CK, the application of compound aqueous solutions increased the Shannon and Simpson indices of bacteria and the Chao, Shannon, and Simpson indices of fungi, with the most pronounced effects observed at higher concentrations. These findings suggest that BAE and its compound aqueous solutions enhance the α-diversity of bacterial and fungal communities, with a greater impact on fungi than bacteria.

#### 3.3.2 Effects on soil microbial abundance

**Figure 4:**
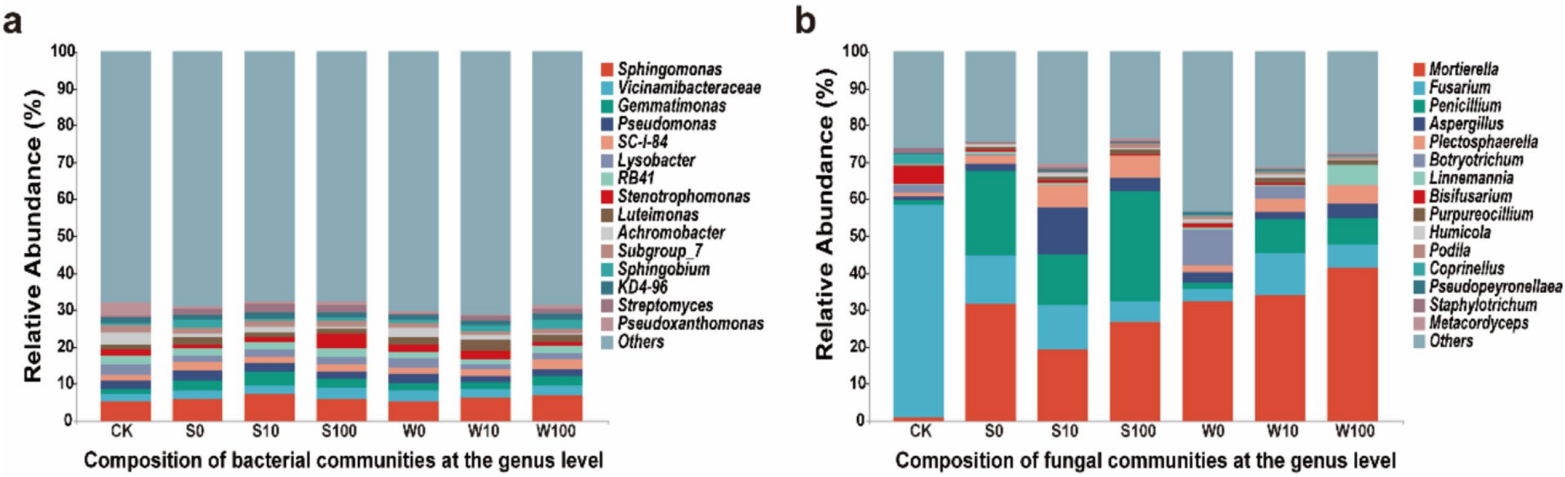
The abundance of bacterial **(a)** and fungal **(b)** community composition in rhizosphere soil of flue-cured tobacco under different treatments.

Analysis of soil microbial community composition revealed that the dominant bacterial genera were *Sphingomonas*, *Vicinamibacteraceae*, *Gemmatimonas*, *Pseudomonas*, and *SC-I-84*. Among these, *Sphingomonas*, *Vicinamibacteraceae*, and *Gemmatimonas* exhibited their highest abundances in the S10 treatment, showing increases of 36.97%, 34.59%, and 263.99% compared to CK, respectively (Figure 4a). In the fungal community, the major dominant genera identified were *Mortierella*, *Fusarium*, *Penicillium*, *Plectosphaerella*, and *Botryotrichum*. The application of BAE and compound aqueous solutions significantly increased the abundance of *Mortierella* and *Penicillium*, while reducing the abundance of *Fusarium* (Figure 4b). These findings suggest that BAE and compound aqueous solutions altered the soil microbial community structure, with a more pronounced effect on the fungal community than on the bacterial community. Additionally, the impact of BAE on the fungal community was greater than that of the compound aqueous solutions.

### 3.4 Effects of BAE and the compound aqueous solutions on soil microbial function

**Figure 5:**
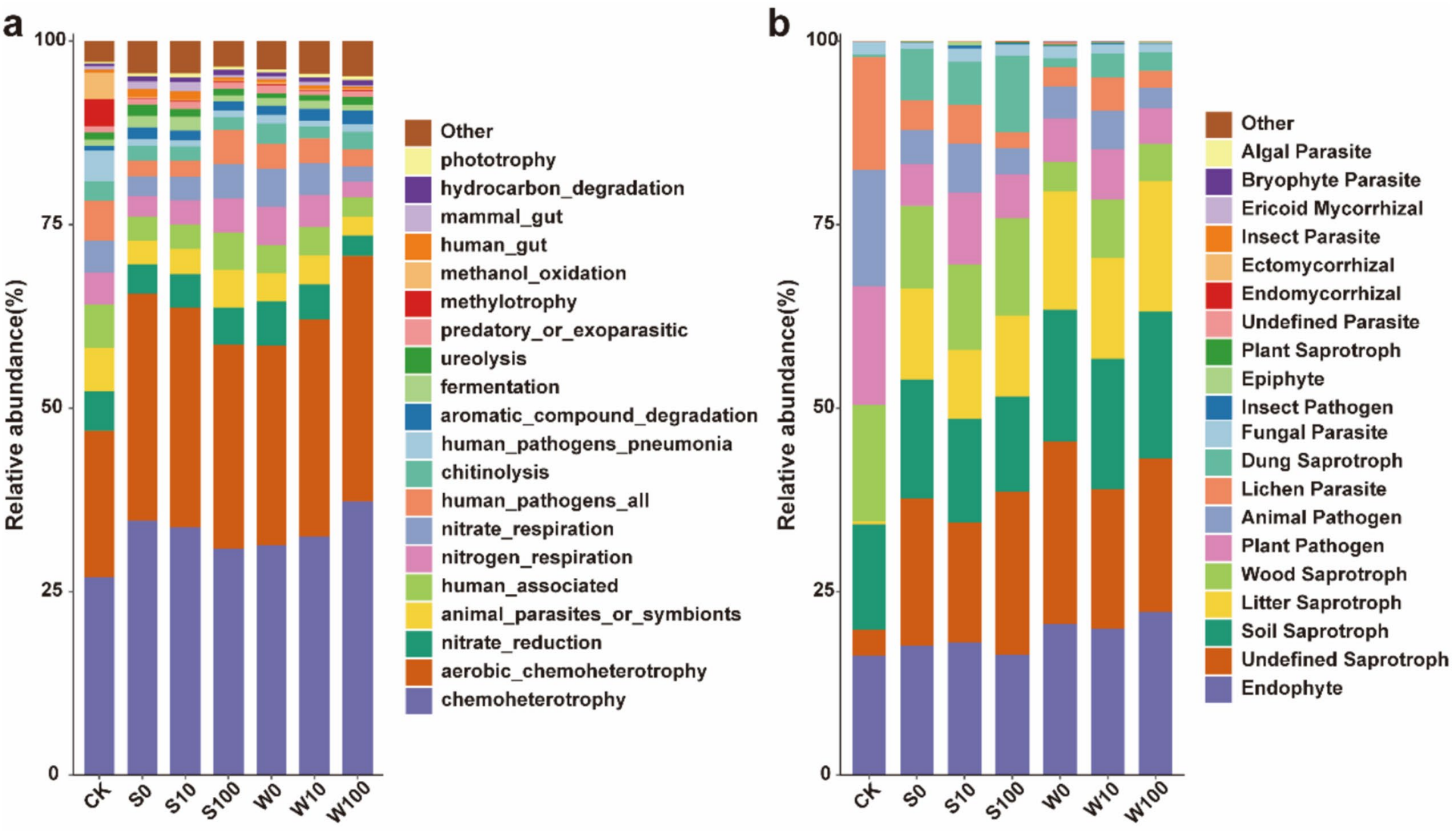
Functional diversity histogram of bacteria **(a)** and fungal **(b)** in rhizosphere soil of flue-cured tobacco under different treatments.

The ecological functions of bacteria were predicted using the FATROTAX database. The results indicated that the application of BAE and its compound aqueous solutions influenced 70 functions in the rhizosphere soil of tobacco. These functions primarily included Chemoheterotrophy, Aerobic-chemoheterotrophy, Nitrate-reduction, Nitrogen-respiration, Nitrate-respiration, Chitinolysis, and Aromatic-compound-degradation (Figure 5a; Supplementary Table S3). The both treatments significantly increased the relative abundance of functional bacteria involved in Chemoheterotrophy, Aerobic-chemoheterotrophy, and Aromatic-compound-degradation, while decreasing the relative abundance of bacteria associated with Nitrate-reduction, Nitrogen-respiration, and Nitrate-respiration.

Functional predictions using the FUNGuild database identified three primary fungal functions in rhizosphere soil: Saprotroph, Pathogen, and Parasite (Figure 5b; Supplementary Table S4). The application of BAE and their compound solutions increased the relative abundance of functional fungi classified as Undefined Saprotroph, Soil saprotroph, Litter saprotroph, and Dung saprotroph. Notably, the relative abundance of Undefined saprotroph, Soil saprotroph, and Litter saprotroph was higher in the compound aqueous solution treatment compared to the BAE treatment, whereas the opposite trend was observed for Dung saprotroph. Conversely, both treatments reduced the relative abundance of functional fungi, including Wood saprotroph, Plant pathogen, Animal pathogen, and Lichen parasite, with a more pronounced effect in the acid extract treatment group. These results indicate that applying BAE and its compound aqueous solution enhances Saprotroph functionality while reducing Pathogen and Parasite functions.

### 3.5 Co-occurrence network analysis of soil microbial communities in the rhizosphere

**Figure 6:**
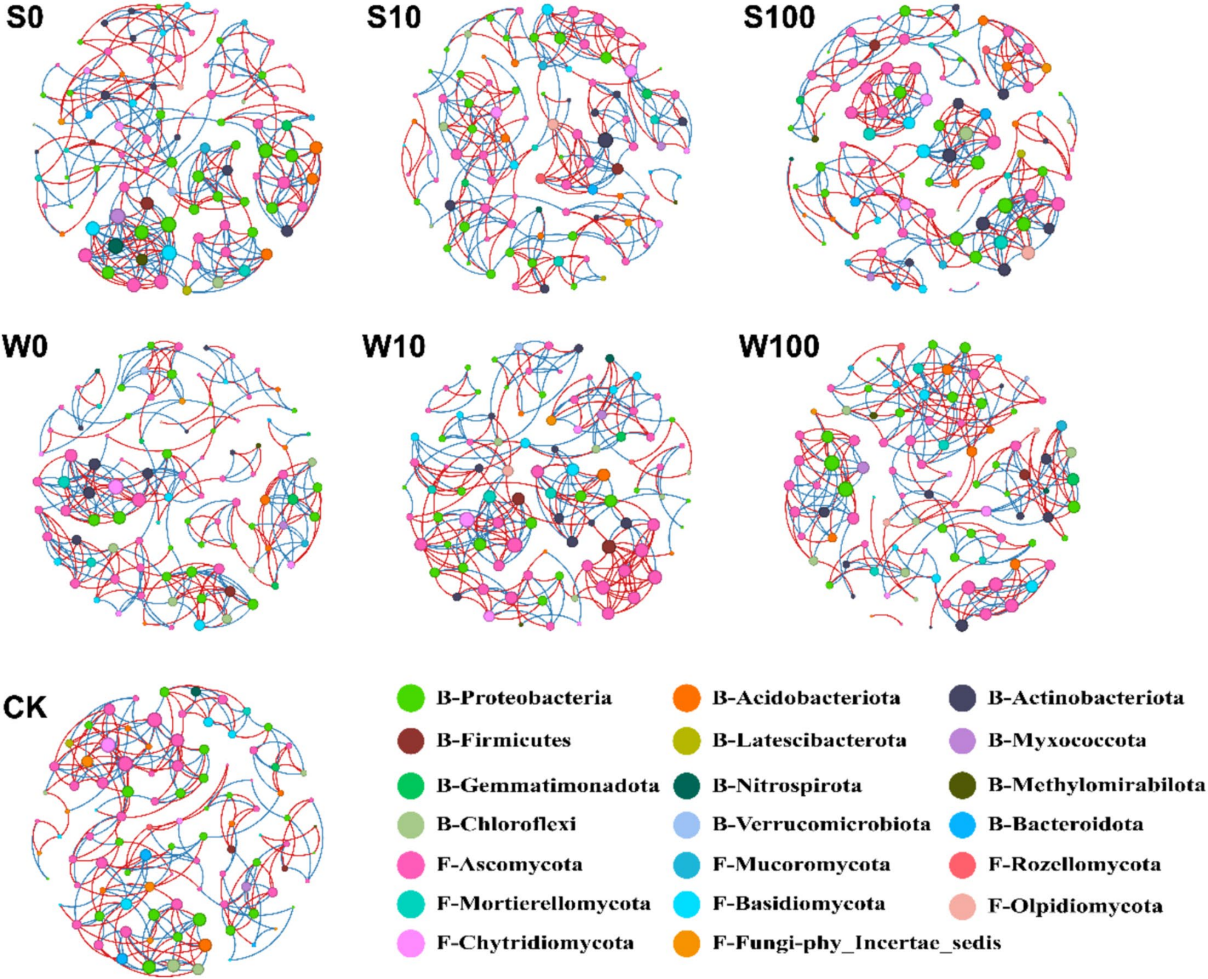
Co-occurrence networks of the microbial communities in the different treatments. Connections represent a statistically significant (*p* < 0.01) strong positive correlation (red, Spearman’s *p* > 0.6) or negative correlation (blue, Spearman’s *p* < −0.6). The “B” and “A” in front of the node legend represent bacteria and fungi, respectively. Size of each node is proportional to the number of connected edges, nodes of the same color belong to the same gate, and the thickness of each connection between two nodes is proportional to the value of the Spearman correlation coefficient *p* > 0.6 or *p* < −0.6.

The co-occurrence network of bacteria and fungi primarily reflects species coexistence relationships within the samples, with nodes and edges representing the scale and complexity of these interactions. A co-occurrence network analysis of the 50 most abundant bacterial and fungal genera in rhizosphere soil (Figure 6) is summarized in Table 2. The analysis revealed that the main bacterial and fungal nodes in the rhizosphere soil belonged to 12 bacterial phyla and 8 fungal phyla, predominantly B-Proteobacteria, B-Gemmatimonadota, F-Ascomycota, and F-Basidiomycota. Compared to the control group (CK), the BAE treatment increased the average clustering coefficient, average degree, and positive correlation ratio of the microbial network structure in the rhizosphere soil while reducing the average path length and network diameter. This indicates that the BAE treatment enhances the complexity of the microbial network structure, with complexity increasing at higher dilution rates. The compound aqueous solutions treatment also improved microbial network complexity compared to CK, with the highest positive correlation ratio, modularity, and average clustering coefficient observed at a 10-fold dilution, resulting in a more complex network structure. These findings suggest that both BAE and its compound aqueous solutions enhance microbial network complexity in the rhizosphere, with greater complexity observed under BAE treatment than compound solutions treatment. Furthermore, in the S100, W0, and W100 treatments, the positive correlation ratios exceeded the negative correlation ratios, thereby promoting mutualistic relationships between bacteria and fungi.

**Table 2:** Topological parameters of soil microbial co-occurrence network under different treatments.

Treatments	CK	S0	S10	S100	W0	W10	W100
Modularity (MD)	0.814	0.807	0.838	0.848	0.817	0.840	0.836
Average clustering coefficient	0.697	0.770	0.757	0.815	0.698	0.788	0.753
Average path length	5.693	9.414	3.131	2.102	5.611	3.583	3.176
Network diameter	19	29	9	7	18	11	10
Average degree (AD)	4.940	5.567	5.071	5.414	5.091	5.271	4.788
Positive	124	123	126	140	123	137	122
Negative	123	147	125	128	129	116	115
Nodes	100	97	99	99	99	96	99
Edges	247	270	251	268	252	253	237

### 3.6 Relationship between agrochemical properties of tobacco rhizosphere soil and soil microbial communities

**Figure 7:**
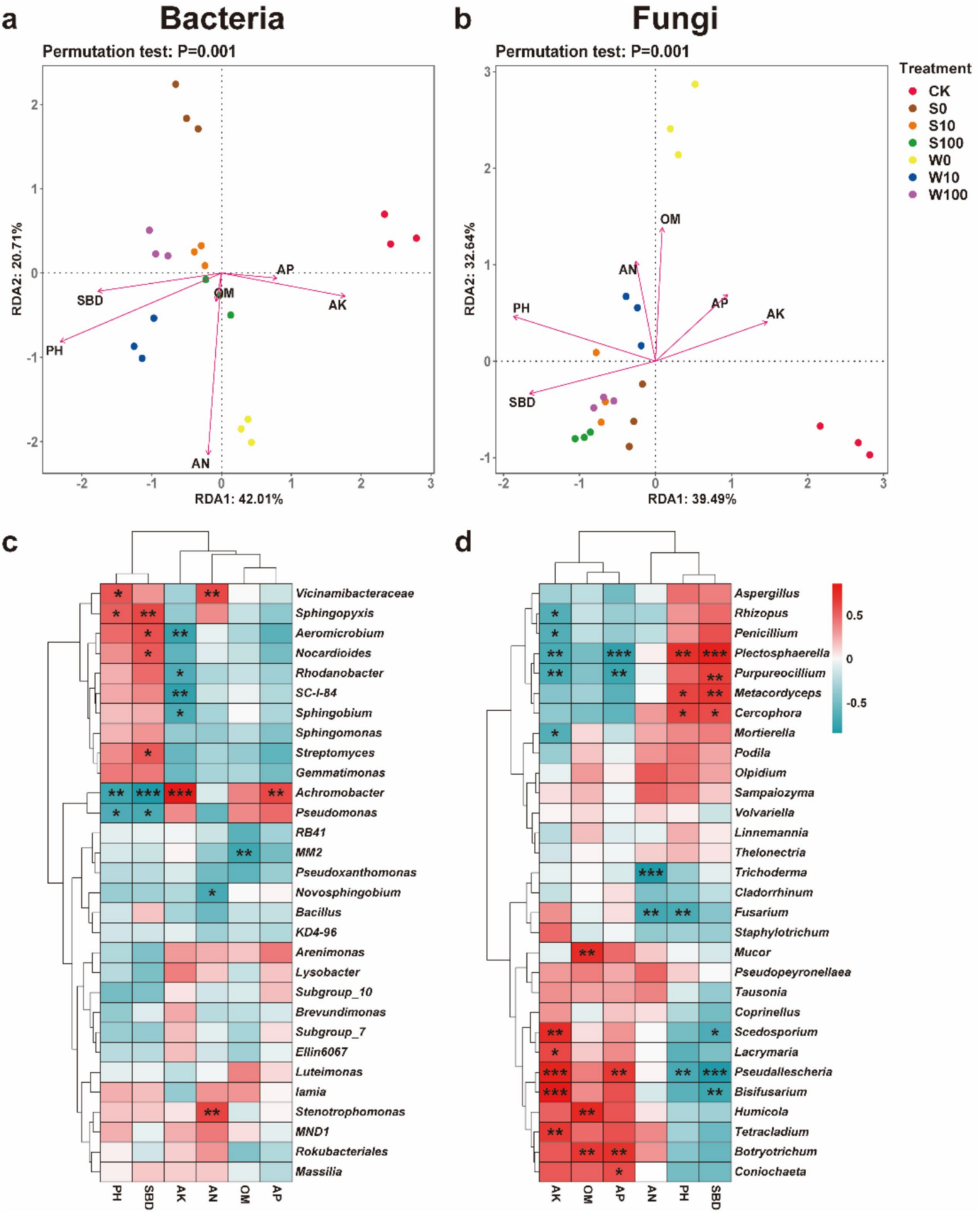
Redundancy analysis and Pearson analysis of the microflora and soil properties in the rhizosphere. Relationships between soil physicochemical properties and relative abundances of the top 30 dominant genera of bacteria **(a,c)** and fungi **(b,d)**. ^*^p < 0.05; ^**^p < 0.01; ^***^p < 0.001.

The relationship between the agrochemical properties of soil and the overall microbial community structure was analyzed using Redundancy Analysis (RDA). The results showed that treatment with BAE and its compound aqueous solutions influenced the bacterial community structure in tobacco rhizosphere soil (Figure 7a), explaining 42.01% and 20.71% of the variability through RDA1 and RDA2, respectively. The environmental factors affecting the bacterial community were ranked as follows: AN > pH > SBD > AK > AP > OM. For the fungal community structure in the rhizosphere soil (Figure 7b), RDA1 and RDA2 accounted for 39.49 and 32.64% of the variability, respectively. Among the environmental factors, SBD, pH, and OM had the most pronounced effects on the fungal community, followed by AN, AP, and AK.

The relationship between dominant microbial genera and soil agrochemical properties was further analyzed using Pearson correlation coefficients. In the bacterial community (Figure 7c), *Achromobacter*, *Vicinamibacteraceae*, *Sphingopyxis*, and *Aeromicrobium* are key microorganisms that affect soil physicochemical properties, and significantly regulate soil pH, SBD, AK, and AN. Notably, *Achromobacter* exhibited a highly significant positive correlation with AK and AP, but a highly significant negative correlation with pH and SBD. *Vicinamibacteraceae* was significantly positively correlated with AN, while *Aeromicrobium* showed a highly significant negative correlation with AK. Additionally, *Sphingopyxis* had a highly significant positive correlation with SBD. In the fungal community (Figure 7d), *Pseudallescheria*, *Plectosphaerella*, *Purpureocillium*, *Bisifusarium*, and *Fusarium* were the primary genera influencing soil nutrients. The soil parameters AK, pH, AP, and SBD were notably affected by these fungi. *Plectosphaerella* and *Purpureocillium* demonstrated significant positive correlations with SBD and highly significant negative correlations with AK and AN. *Fusarium* showed a highly significant negative correlation with AN, whereas *Pseudallescheria* was highly positively correlated with AK and AP but negatively correlated with pH and SBD. *Bisifusarium* displayed a highly significant positive correlation with AK and a highly significant negative correlation with SBD. These findings suggest that shifts in soil microbial community structure by BAE and its compound aqueous solutions can improve soil agrochemical properties.

### 3.7 Correlation analysis of agronomic traits and biomass of tobacco with soil agrochemical properties and microbial communities

**Figure 8:**
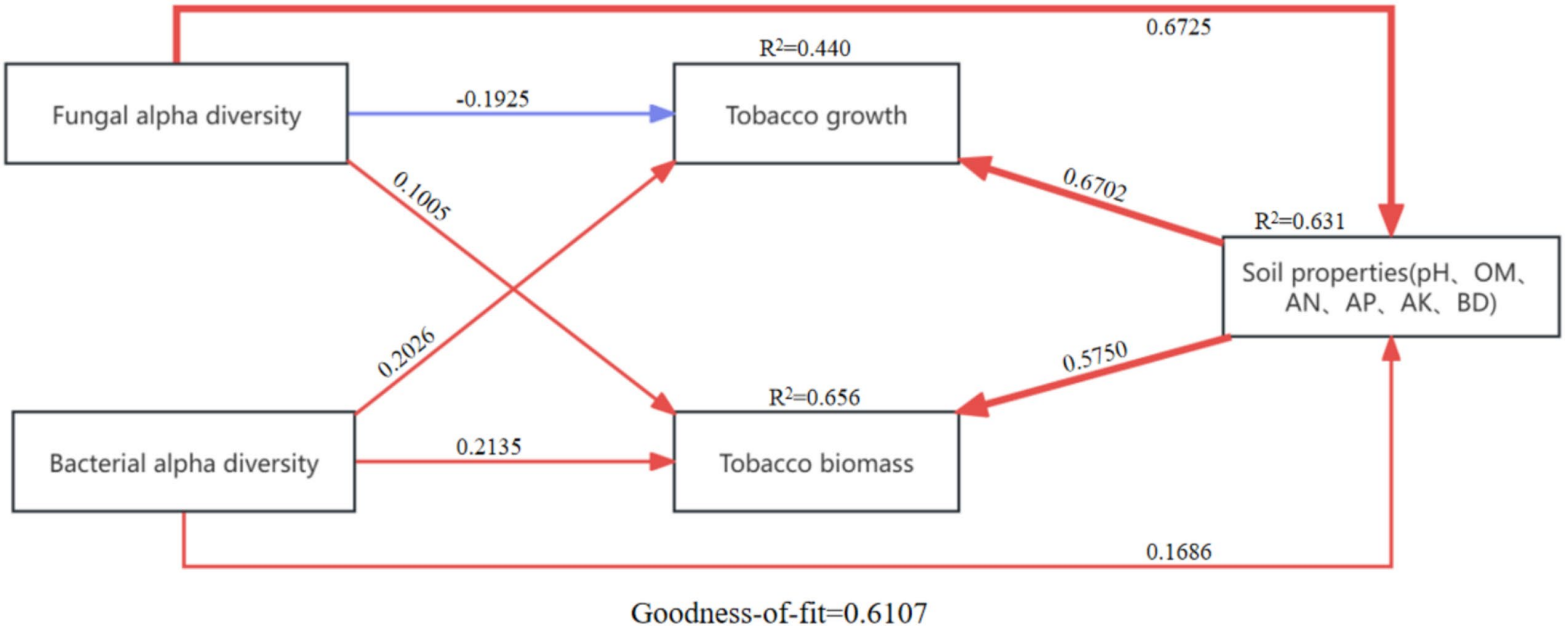
Partial least squares path model (PLSPM) of fungal alpha diversity, bacterial alpha diversity, tobacco growth, tobacco biomass and soil properties. The width of the arrow is proportional to the strength of the path coefficient. The blue and red arrows indicate the positive and negative flow of causality, respectively (*p* < 0.05). The number on the arrow indicates the effective normalized path coefficient. R^2^ represents the variance of the dependent variable explained by the model.

The relationships among agronomic traits, tobacco biomass, soil agrochemical properties, and microbial communities were analyzed using Partial Least Squares Path Modeling (PLS-PM) (Figure 8). The results showed that fungal *α*-diversity positively influenced soil agrochemical properties (0.6725) and tobacco biomass (0.1005) but negatively affected agronomic traits (−0.1925). In contrast, bacterial α-diversity had positive effects on tobacco biomass (0.2135), agronomic traits (0.2026), and soil agrochemical properties (0.1686). Furthermore, soil agrochemical properties positively influenced both agronomic traits (0.6702) and biomass of tobacco (0.5750). These findings underscore the significant positive regulatory capacity of soil microbes in improving soil agrochemical properties, thereby enhancing tobacco growth.

## 4 Discussion

### 4.1 Effects of different treatments on soil agrochemical properties and microbial diversity

Nutrient content is crucial for maintaining soil fertility within ecosystems (Mitsuta et al., 2025). The application of biochar improves soil micro-ecology by regulating pH and the carbon-to-nitrogen (C/N) ratio (Muqiu et al., 2023; Myung et al., 2024), enhancing nutrient content, reducing SBD, and promoting aggregate formation, which jointly promote nutrient uptake by plants (Gao et al., 2017). As a rich source of OM, biochar contains high levels of phenolic and carboxylic carbon, effectively increasing soil OM content (Hosseini et al., 2024; Kassa et al., 2025). In this study, the application of high concentrations of BAE and its compound aqueous solutions significantly elevated soil OM, pH, and AN level. Furthermore, soil pH and AN exhibited a highly significant positive correlation with the *Vicinamibacteraceae* bacterial genus, which propagate rapidly in neutral soils and participate in nitrogen cycling, thereby promoting nutrient availability and maintaining soil ecological balance (Liu et al., 2024). Additionally, biochar’s alkaline nature, porous structure, and high carbon content also contributed to an increased ratio of soil C/N, improving nitrogen adsorption and fixation (Yuan et al., 2023; Zheng et al., 2020). Consistent with these findings, previous studies have demonstrated that soil microbial diversity strongly influences soil pH. Within specific ranges, microbial diversity positively correlates with soil pH (Johannes and Erland, 2009). For example, in rice paddy soils, microbial diversity indices showed a significant positive relationship with pH (Zheng et al., 2016). Similarly, in red soils, bacterial Shannon and Chao1 indices positively correlate with pH and AP content while negatively correlating with the content of AN and AK (Wu et al., 2020). The application of BAE and compound aqueous solutions reduced AK and AP contents, likely due to the increased relative abundance of the fungal genus *Mortierella*, which is saprophytic (Markovina et al., 2005; Lumley et al., 2001) and are able to release organic acids that dissolve soil phosphorous (Osorio and Hante, 2014), enhancing its availability to plants. Interestingly, the application of BAE and compound aqueous solutions increased SBD, contrasting with earlier studies (Tianbao et al., 2021). This increase may be attributed to significant variations in the physicochemical properties of biochar derived from different sources. Additionally, acid extraction optimized the specific surface area and porosity of biochar, promoting micro-aggregate formation and thereby increasing SBD (Malik et al., 2025). These findings suggest that application of BAE and its compound aqueous solutions dynamically modify soil microbial community structure and function, ultimately enhancing soil nutrient profiles.

Soil microbes enhance and sustain the dynamic balance of the soil environment through their metabolic activities, thereby promoting plant growth (Bonkowski et al., 2009). Studies indicate that biochar application improves the abundance and community structure of dominant soil microbes (Kang et al., 2025). In the present study, the application of BAE and its compound aqueous solutions altered the relative abundance and diversity of soil microbial communities, caused the up-regulation of microbial *α*-diversity (Figures 2, 3). This effect was more pronounced in fungal α-diversity compared to bacterial α-diversity, likely due to the concentration of the acidic extract and associated changes in soil physicochemical properties (Fei et al., 2020). Additionally, the observed increase in soil pH was more favorable for fungal survival (Cong et al., 2021). Furthermore, the application of the BAE reduced the relative abundance of the *Streptomyces* genus, the largest genus within the phylum Actinobacteria, which plays a dominant role in soil ecosystems. *Streptomyces* exhibits inhibitory effects on fungi and has potential applications in biological control strategies for plant diseases (Munusamy et al., 2022). Its reduced abundance may contribute to the observed increase in fungal community diversity. Moreover, the application of both the BAE and the compound aqueous solutions increased the relative abundances of bacterial genera such as *Gemmatimonas*, *Sphingomonas*, *Pseudomonas*, and *Vicinamibacteraceae*, as well as fungal genera including *Mortierella*, *Fusarium*, *Penicillium*, and *Aspergillus*. Notably, *Gemmatimonas* can convert N_2_O in the atmosphere into N_2_, and its survival in soil helps reduce N_2_O emissions into the atmosphere (Mamoru et al., 2022), *Sphingomonas* can produce various specific enzymes such as hydrolytic enzymes and oxidases, with a wide range of metabolic diversity. It can survive and function in multiple environments, making it an important organic compound degrader in soil (Manman et al., 2023). *Pseudomonas* is known for suppressing pathogen growth and degrading pollutants (Huber and Overmann, 2018), and *Vicinamibacteraceae* contributes to soil nitrogen cycling and nutrient balance. Among fungi, *Mortierella* can produce and release organic acids during its metabolic process. Organic acids can form complexes with metal ions on the surface of insoluble phosphate minerals in soil. In addition, the release of organic acids can lead to a decrease in pH in the soil environment, Acidic environments help to disrupt the complexation between phosphate minerals and soil particles, further promoting phosphate dissolution (Osorio and Hante, 2014). The enrichment of *Penicillium* and *Fusarium* plays a role in regulating soil pH, with *Penicillium* specifically releasing phosphatases and phytases to enhance phosphorus cycling in the soils (Kang et al., 2021). Additionally, *Aspergillus* can convert phosphorus and potassium into soluble forms, making them more accessible to plants (Wang et al., 2024). These findings suggest that the application of BAE and compound aqueous solutions improves the structure and diversity of soil microbial communities.

### 4.2 Interaction between soil agrochemical properties and microbial diversity

The structure of soil microbial communities and soil agrochemical properties are mutually reinforcing (Fei et al., 2020). In this study, the application of BAE and its compound aqueous solution increased the relative abundances of functional bacteria such as Chemoheterotrophy, Aerobic-chemoheterotrophy, and Aromatic-compound-degradation. Specifically, Chemoheterotrophy and Aerobic-chemoheterotrophy bacteria play pivotal roles in modulating various forms of carbon content and facilitating carbon cycling. Additionally, the enhanced abundance of the Aromatic-compound-degradation group improves the degradation of phenolic allelochemicals present in the root exudates of continuously cultivated of flue-cured tobacco (Susilowati et al., 2015). The application also increased the relative abundance of Saprotroph fungi while reducing Pathogen and Parasite fungi. This reduction of Pathogen and Parasite fungi reduced the incidence of fungal diseases, thereby supporting tobacco growth. Saprotroph fungi, as primary decomposers of soil OM (Xiaoli et al., 2022), showed a positive correlation with higher OM content. These results demonstrate that the relative abundances of functional microbial groups are significantly correlated with soil nutrient dynamics. By promoting the abundance of beneficial microbial groups, BAE and its compound aqueous solution improve soil nutrient content and promoting tobacco growth.

Symbiotic networks are commonly used to assess potential interactions within microbial communities (Samiran et al., 2019). In this study, the application of BAE and its compound aqueous solution enhanced correlations among soil microbes (Figure 6; Table 2). This effect can be attributed to increased microbial abundance and *α*-diversity, which enable microbial communities to form more complex and cohesive symbiotic networks that better adapt to environmental changes induced by biochar application (Yanan et al., 2023). Following the application of the BAE and compound aqueous solution, the dominant bacterial phyla were Proteobacteria, Gemmatimonadota, Actinobacteria, and Acidobacteriota. Proteobacteria is a widely distributed bacterial community in soil, which includes many members capable of nitrogen fixation. They can convert atmospheric nitrogen into a form that plants can absorb, thereby promoting plant growth (Wang et al., 2025). Gemmatimonadota can secrete acid phosphatase in soil to decompose organic phosphorus and promote plant uptake of phosphorus (Han et al., 2024). Actinobacteria, typically found in neutral to mildly alkaline soils, play a crucial role in decomposing insoluble OM, such as cellulose and chitin, and in the mineralization of soil nutrients (Wu et al., 2020). These bacteria are also integral to soil nitrogen cycling, acting as a “fertilizer factory” that supplies essential nitrogen for plant growth (Mitsuta et al., 2025). In contrast, Acidobacteriota, which thrive under oligotrophic conditions, are often enriched in nutrient-poor soils and are associated with the mineralization of soil OM (Li M. et al., 2024). Thus, changes in the structure of soil microbial communities significantly affect soil nutrient dynamics and pH balance.

Previous studies have indicated that Proteobacteria is distributed group of bacteria in soil, included many members capable of nitrogen fixation, converted atmospheric nitrogen into a form usable by plants and thus promoted plant growth. Moreover, they participate in the decomposition of organic matter and nutrient cycling, which significantly impacts soil health and plant productivity.

### 4.3 Soil microbes enhance tobacco growth by regulating soil nutrients

Soil microbial communities play a pivotal role in regulating soil nutrient content, thereby promoting tobacco growth (Sasse et al., 2018). Partial Least Squares Path Modeling (PLS-PM) analysis demonstrates that the application of BAE and its aqueous solutions identifies soil microbes as primary determinants of soil agrochemical properties (Figure 8). These microbes promote tobacco growth by enhancing the availability of essential soil nutrients. Increases in soil OM and AN supply critical carbon and nitrogen sources for microbial survival, influencing microbial community structure and diversity. Fungi also contribute to the regulation of soil carbon and nitrogen sources (Marchal et al., 2025). Consistent with these findings, bacterial genera such as *Sphingomonas* and *Streptomyces* have been shown to increase ammonium nitrogen levels, total potassium, and the activities of enzymes like urease, catalase, and saccharase, thereby boosting strawberry plant growth and yield (Li et al., 2025). Similarly, biochar application reportedly enhances the abundance of Acidobacteriota, which improves soil pH, OM content, and alkali-soluble nitrogen levels (Mitsuta et al., 2025). Interactions among beneficial rhizobacteria and other microbial communities further enrich soil nutrient content, facilitating crop growth (Neemisha et al., 2022). Collectively, these findings highlight soil microbial diversity as a critical driver of soil agrochemical properties, establishing a strong positive correlation between the two.

## 5 Conclusion

The application of BAE and compound aqueous solutions increased the soil microbial community structural diversity and the relative abundance of beneficial functional groups, thereby enhancing the complexity and stability of the soil microbial network. This intervention also elevated soil pH, available nitrogen, and organic matter content while reducing available potassium and phosphorus content, thereby balancing soil nutrient content. Consequently, these improvements promoted the growth and dry matter accumulation of flue-cured tobacco, thereby providing a theoretical framework for understanding how biochar-derived soluble small molecular compounds impacted tobacco growth and soil nutrient utilization. The study found no significant differences in the effects of BAE and compound aqueous solutions on flue-cured tobacco and soil, indicating the feasibility of using compound aqueous solutions. Therefore, this finding provides a technical foundation and valuable insights for the development and application of related products of BAE and compound aqueous solutions.

## Data Availability

Microbial data associated with this article can be found in the online version. Raw sequencing data were deposited in the NCBI Sequence Read Archive (SRA, Bacteria: https://www.ncbi.nlm.nih.gov/sra/PRJNA1240441; Fungi: https://www.ncbi.nlm.nih.gov/sra/PRJNA1240246) with Accession No. PRJNA1240441 and PRJNA1240246.
