# Supplementary material for: The phosphoric acid extract of fresh biochar and its compound aqueous solutions promoted tobacco plant growth by regulating nutrient-related microorganisms in rhizosphere soil
Source: Front Microbiol. 2025 May 22;16:1601567. doi: 10.3389/fmicb.2025.1601567 (PMC12142071; doi:10.3389/fmicb.2025.1601567)
Supplement: Supplementary file 1 [file Data_Sheet_1.pdf]

SUPPLEMENTARY INFORMATION

Table S1 The agronomic traits of flue-cured tobacco plant under different treatments.

	Treatment	Number of leaves	Plant height（cm）	Internode length（cm）	Stem girth（cm）	Maximum leaf length（cm）	Maximum blade width（cm）
Resettling stage	CK	9.67±0.67b	13.53±0.03c	2.40±0.21d	5.73±0.13c	41.00±1.53d	22.50±0.29c
	S0	11.00±0.67ab	15.49±0.03a	3.07±0.07bc	6.87±0.03b	49.67±0.17a	23.67±0.17b
	S10	10.00±0.33b	14.17±0.12bc	3.07±0.03bc	6.73±0.03b	47.67±0.17abc	22.83±0.17c
	S100	9.67±0.67b	14.23±0.53bc	2.47±0.09d	5.90±0.06c	46.33±0.17c	22.67±0.17c
	W0	11.67±0.33a	16.23±0.27a	3.60±0.06a	7.27±0.07a	49.00±0.58ab	25.17±0.17a
	W10	11.00±0.33ab	14.55±0.04b	3.27±0.12ab	7.13±0.03a	47.33±0.17bc	23.67±0.17b
	W100	10.67±0.33ab	14.56±0.11b	2.90±0.06c	6.77±0.09b	46.50±0.29c	22.50±0.29c
Vigorous growing stage	CK	18.00±0.00bc	104.67±0.17f	4.97±0.09ab	8.27±0.18b	68.33±1.20b	26.83±0.44c
	S0	19.00±0.58b	109.67±0.17c	5.43±0.15a	9.07±0.30a	70.00±0.58ab	27.33±0.88bc
	S10	18.00±0.58bc	109.83±0.17c	4.33±0.33bc	8.43±0.07b	69.00±0.58ab	27.00±0.50c
	S100	17.67±0.33bc	106.83±0.17e	4.23±0.15c	8.10±0.06b	68.00±1.53b	25.83±0.44c
	W0	20.67±0.33a	116.67±0.17a	5.43±0.22a	9.53±0.23a	72.33±1.64a	30.17±0.93a
	W10	18.00±0.00bc	110.83±0.17b	4.73±0.19bc	8.50±0.15b	69.83±0.60ab	29.00±0.50ab
	W100	17.00±0.58c	108.83±0.17d	4.43±0.20bc	8.30±0.10b	68.33±0.73b	25.67±0.33c
Upper leaves mature stage	CK	/	105.67±0.17f	5.48±0.21c	8.07±0.07c	78.33±0.44b	27.50±0.67a
	S0	/	119.13±0.19a	6.77±0.15a	9.93±0.54a	83.67±0.73a	30.33±0.58a
	S10	/	110.50±0.29b	6.43±0.07ab	9.23±0.43abc	80.83±0.73b	30.17±1.86a
	S100	/	107.50±0.29d	5.52±0.14c	8.60±0.58bc	79.83±0.60b	27.83±0.33a
	W0	/	110.67±0.17b	5.97±0.09bc	9.67±0.44ab	80.33±1.36b	28.83±1.00a
	W10	/	109.50±0.29c	5.73±0.37c	9.10±0.49abc	80.00±1.04b	27.83±0,33a
	W100	/	106.50±0.29e	5.37±0.23c	8.23±0.23c	79.50±0.76b	27.50±0.33a

Table S2 Microbial diversity index under different treatments.

	Treatments	CK	S0	S10	S100	W0	W10	W100
Bacterial	Chao1	3469.01±43.17bc	3718.96±181.44ab	3727.30±5.39ab	3902.66±106.91a	3733.57±68.50ab	3167.31±59.63d	3395.06±85.29cd
	Shannon	10.32±0.02c	10.62±0.08ab	10.63±0.01ab	10.68±0.02a	10.66±0.03ab	10.39±0.03c	10.52±0.06b
	Simpson	1.00±0.00d	1.00±0.00a	1.00±0.00ab	1.00±0.00c	1.00±0.00ab	1.00±0.00c	1.00±0.00bc
Fungal	Chao1	404.72±2.79d	452.34±24.17cd	572.65±27.93a	489.25±21.34bc	614.24±42.28a	552.89±9.76ab	588.65±14.46a
	Shannon	4.46±0.10e	4.87±0.08de	5.81±0.19b	4.79±0.02de	6.39±0.27a	5.44±0.15bc	5.04±0.15cd
	Simpson	0.84±0.01d	0.93±0.00ab	0.95±0.01a	0.90±0.01bc	0.95±0.01a	0.90±0.01bc	0.87±0.02cd

Table S3 Bacteria functional diversity under different treatments.

Treatments	nitrate_respiration	nitrate_reduction	nitrogen_respiration	photosynthetic_cyanobacteria	oxygenic_photoautotrophy	photoheterotrophy	phototrophy	ureolysis	chemoheterotrophy
CK	2.83±0.05ab	3.52±0.06a	2.84±0.05ab	0.04±0.80bc	0.04±0.80bc	0.10±0.19d	0.14±0.24c	0.63±0.12b	17.67±0.13c
S0	1.60±0.11c	2.36±1.72c	1.63±0.10c	0.01±0.20d	0.01±0.20d	0.26±0.24ab	0.27±0.24b	0.91±0.18a	20.42±0.06a
S10	1.94±0.09c	2.71±2.70bc	1.97±0.01c	0.05±0.20b	0.05±0.20b	0.31±0.26a	0.35±0.28a	0.65±0.39b	20.22±0.37a
S100	2.84±0.14ab	3.10±1.91ab	2.87±0.14ab	0.07±0.01a	0.07±0.01a	0.19±0.11c	0.26±0.10b	0.55±0.36ab	18.82±0.19b
W0	3.04±0.23a	3.51±2.53a	3.06±0.23a	0.03±0.20c	0.03±0.20c	0.23±0.04bc	0.25±0.05b	0.38±0.19d	18.44±0.14bc
W10	2.52±0.15b	2.77±1.72bc	2.55±0.15b	0.00±0.10d	0.00±0.10d	0.27±0.11ab	0.27±0.13b	0.46±0.73cd	18.93±0.21b
W100	1.11±0.02d	1.50±2.70d	1.14±0.02d	0.01±0.50d	0.01±0.50d	0.29±0.12a	0.30±0.09ab	0.59±0.38b	20.12±0.54a

Table S4 Fungal functional diversity under different treatments.

Treatments	DungSaprotroph	Endomycorrhizal	Litter Saprotroph	Plant Pathogen	Soil Saprotroph	Undefined Saprotroph	Wood Saprotroph
CK	0.38±0.04e	0.00±0.02b	0.33±0.00e	15.51±0.05a	13.72±0.30cd	3.41±0.14e	15.15±0.05a
S0	6.71±0.06b	0.03±0.07b	11.90±0.11bc	5.46±0.65cd	15.56±0.47bc	19.28±1.04b	10.8±0.71bc
S10	5.16±0.24c	0.01±0.07b	8.35±0.05d	8.58±0.66b	12.45±0.15d	14.46±0.40d	10.26±0.37c
S100	9.81±0.51a	0.12±0.48a	10.43±0.10cd	5.69±0.55cd	12.30±0.78d	21.01±0.58ab	12.54±0.79b
W0	0.98±0.13e	0.02±0.06b	13.72±0.15b	5.08±0.49cd	15.23±1.14bc	21.18±0.29a	3.36±0.34e
W10	2.97±0.45d	0.01±0.02b	12.56±0.10bc	6.22±0.47c	16.32±0.49b	17.47±0.75c	7.36±0.99d
W100	2.33±0.44d	0.02±0.01b	16.69±0.05a	4.50±0.13d	18.67±0.41a	19.59±0.11ab	4.70±0.57e
